# TUNEL Assay: A Powerful Tool for Kidney Injury Evaluation

**DOI:** 10.3390/ijms22010412

**Published:** 2021-01-02

**Authors:** Christopher L. Moore, Alena V. Savenka, Alexei G. Basnakian

**Affiliations:** 1 Department of Pharmacology & Toxicology, University of Arkansas for Medical Sciences, 4301 West Markham Street, #638, Little Rock, AR 72205, USA; CMoore9@uams.edu (C.L.M.); SavenkaAlenaV@uams.edu (A.V.S.); 2 John L. McClellan Memorial VA Hospital, Central Arkansas Veterans Healthcare System, 4300 West 7th Street, Little Rock, AR 72205, USA

**Keywords:** TUNEL, cell death, kidney, DNases, endonucleases, tissue injury

## Abstract

Terminal deoxynucleotidyl transferase dUTP nick-end labeling (TUNEL) assay is a long-established assay used to detect cell death-associated DNA fragmentation (3’-OH DNA termini) by endonucleases. Because these enzymes are particularly active in the kidney, TUNEL is widely used to identify and quantify DNA fragmentation and cell death in cultured kidney cells and animal and human kidneys resulting from toxic or hypoxic injury. The early characterization of TUNEL as an apoptotic assay has led to numerous misinterpretations of the mechanisms of kidney cell injury. Nevertheless, TUNEL is becoming increasingly popular for kidney injury assessment because it can be used universally in cultured and tissue cells and for all mechanisms of cell death. Furthermore, it is sensitive, accurate, quantitative, easily linked to particular cells or tissue compartments, and can be combined with immunohistochemistry to allow reliable identification of cell types or likely mechanisms of cell death. Traditionally, TUNEL analysis has been limited to the presence or absence of a TUNEL signal. However, additional information on the mechanism of cell death can be obtained from the analysis of TUNEL patterns.

## 1. Introduction

DNA is the only molecule in the cell that can be repaired, but it cannot be completely resynthesized after damage. DNA destruction is one of the final stages of a multi-step cell death process, which is potentially reversible until the point of terminal DNA fragmentation. Therefore, the latter is a common attribute and mechanistic marker of irreversible cell death [1,2]. Cell death-associated DNA fragmentation is usually “visualized” by the terminal deoxynucleotidyl transferase dUTP nick-end labeling (TUNEL) assay, DNA laddering, or comet assay [3]. Pulse-field electrophoresis [4] and random oligonucleotide-primed synthesis (ROPS) assay [5] are two other rarely used secondary assays. The use of comet assay is limited to in vitro (cultured cells) only, and its quantification is labor-intensive [6]. Performing comet assays in isolated cell nuclei is possible, but it is associated with additional confounding factors during the isolation of nuclei. The second, DNA laddering assay, is not a quantitative method, and the initial hope that the ladder pattern vs. smear would distinguish between apoptosis and necrosis, respectively, was dispelled as this approach proved to be unreliable [7]. Of the three main methods, TUNEL is the most sensitive, least time consuming, and most universally applicable. It can be used in cultured cells and tissues and for all cell death mechanisms. It is accurate, quantitative, easily linked to particular cells or tissue compartments, and can be combined with immunohistochemistry to allow reliable identification of the cell types or mechanisms of cell injury associated with TUNEL-positive signals [2].

DNA breaks measurable by TUNEL are produced mainly by apoptotic endonucleases. The most active endonucleases in the kidney are deoxyribonuclease 1 (DNase I) and endonuclease G (EndoG) [8,9,10]. DNase I is the most active and abundant apoptotic endonuclease in mammals [11]. The kidney, salivary glands, pancreas, and intestine are organs known to produce high amounts of DNase I. Epithelial cells in salivary glands, pancreas, and intestine secrete DNase I as a digestive enzyme in the alimentary tract. Some cellular DNase I fights against “foreign” DNA invading host cells [12]. Non-secreted DNase I presents a hidden danger to host-cell DNA, but normally DNases do not act on host-cell DNA until injury or the death of the cell. In the kidney, DNase I is secreted by tubular epithelial cells, presumably to destroy viruses and bacteria in urine. This function likely takes place in conjunction with proteinases, in particular, meprin, which is the main proteinase in urine [13]. Although it is protective against infection, the presence of highly active DNase in the kidney makes this organ vulnerable to injury, where the activity of DNases is known to be cytotoxic to host cells. DNases promote cell death induced by toxic or hypoxic stimuli and destroy all host kidney DNA cells released as a result of cell death.

Kidneys are filtering organs that remove toxic compounds from the body. High DNase I activity in kidneys makes kidney cells very sensitive to injury from toxic compounds and their metabolic products. This makes TUNEL the most appropriate and applicable method to measure injury to the kidney. In this review, we will focus on TUNEL as a widely used, informative, and precise assay for the measurement of cell death and tissue injury in the kidney. We will discuss aspects of TUNEL as it applies to kidney diseases and injury and identify qualities, techniques, and underused features of TUNEL to promote the productive and appropriate use of this powerful assay in research, diagnostics, and therapy of kidney diseases. We will also discuss various TUNEL image patterns that can be linked to cell death mechanisms. The points raised in this review are broadly applicable for TUNEL use in organs other than the kidney.

## 2. TUNEL Principles

The TUNEL assay was developed in 1992 by Gorczyca et al. [14] and Gavrieli et al. [15], using fluorochrome and avidin-peroxidase labeling, respectively. At that time, there was a desperate need for new methods to assess apoptosis, and TUNEL successfully filled this gap. Initially marketed as an assay for DNA strand breaks during or associated with apoptosis [14,16], in the absence of better assays, TUNEL quickly became the standard assay for apoptosis [17,18]. However, it was recognized almost immediately by researchers that TUNEL indiscriminately measured any DNA fragmentation, not just the one associated with apoptosis [19,20,21]. Nevertheless, the need for apoptotic assays was so great that the rare reports of TUNEL being non-specific for apoptosis were generally ignored. This led to an enormous amount of false-positive reports of apoptosis in the kidney and other organs, to the degree where up to 20% of total cells were reported apoptotic even without injury [22,23,24,25] when changes as small as 0.01% could be statistically significant evidence of tissue injury.

The TUNEL assay has experienced a renaissance in recent years since the end of the boom in apoptosis studies a few years ago. Our PubMed search of journal articles involving “kidney” and “TUNEL” terms produced over 1500 results from 1992 to 2020. In 2019, 170 articles were published, the highest number in a single year over the past 28 years (Figure S1) and which constitute the majority of articles surveyed for this review. Numerous studies used TUNEL-positivity as a universal measure of DNA fragmentation-associated cell death in other types of cell death apart from apoptosis. For example, ferroptosis, a newly discovered form of programmed, non-apoptotic cell death triggered by oxidative damage that occurs during renal ischemia-reperfusion (IR) in mice, can be detected by TUNEL [26]. Pyroptosis, which is an inflammatory form of programmed cell death that is distinct from apoptosis and necrosis, is shown to be associated with TUNEL-positive signals [27,28]. TUNEL-positivity was observed during necroptosis that contributed to the progressive depletion of renal tubule cells in rats subjected to subtotal nephrectomy [29]. In the kidney and other tissues, positive TUNEL signals have been found in necrosis [30,31], dysregulated autophagy [32,33,34,35], anoikis [36,37], mitotic catastrophe [35,38], autolysis [39], paraptosis [40], and aponecrosis [30], demonstrating TUNEL to be a truly universal assay for irreversible cell death.

**Figure 1:**
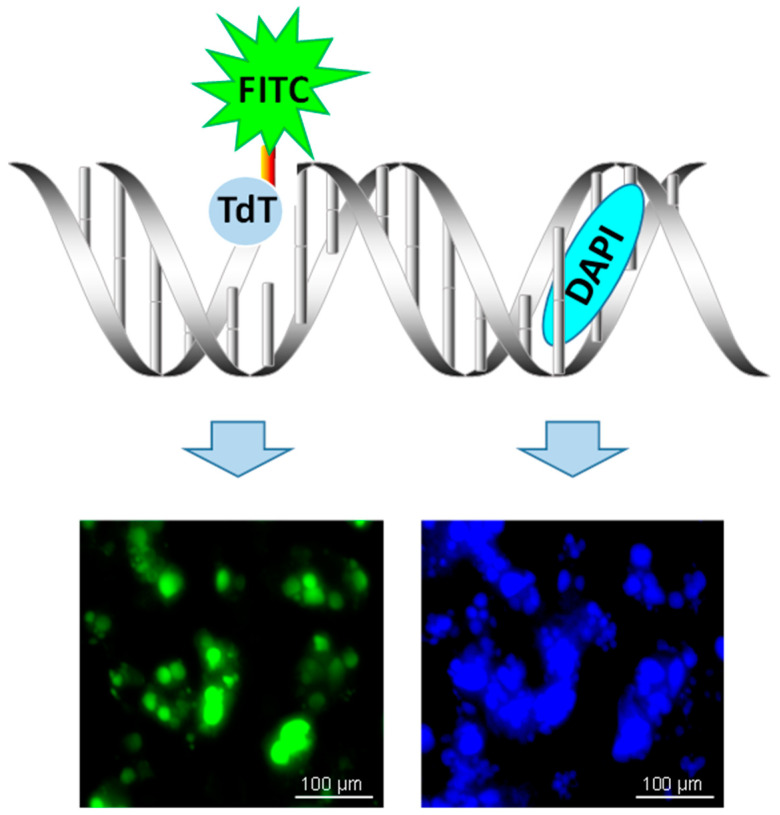
Terminal deoxynucleotidyl transferase dUTP nick-end labeling (TUNEL) assay schematic. Terminal deoxynucleotidyl transferase (TdT) reacts with fluorescein (FITC)-labeled dUTP to attach uridine to 3′-hydroxyl (3′OH) terminus in DNA strand breaks. Double-stranded DNA is counterstained by DAPI that intercalates between strands of double-stranded DNA.

The TUNEL assay is based on labeling of 3′OH ends by a 3′OH-end-specific DNA enzyme, terminal deoxynucleotidyl transferase (TdT) (Figure 1). Use of DNA polymerase or Klenow fragment DNA polymerase in place of TdT may help determine the type of 3′OH ends (nicks, gaps, or overhanging oligos) because DNA polymerases need a template (the opposite DNA strand) and thus will not label hanging or protruding DNA ends. It is important to remember that the production of 3′OH DNA ends is not unique to apoptotic (DNA-degrading) endonucleases. 3′OH DNA ends are a major “communicator” signal, a common denominator of the majority of DNA enzymes. They are produced and used by almost all DNA enzymes in eukaryotes, including DNA repair (apurinic/apyrimidinic, AP) endonucleases, exonucleases, DNA polymerases, DNA ligases, DNA transferases, and topoisomerases. In addition, 3′P, 3′-sugar, or 3′-protein conjugates can be converted to 3′OH termini by phosphatases, deglycosylases, and proteinases, respectively. This is why a precise understanding of what is being measured is necessary for the successful application of TUNEL and interpretation of its results.

**Figure 2:**
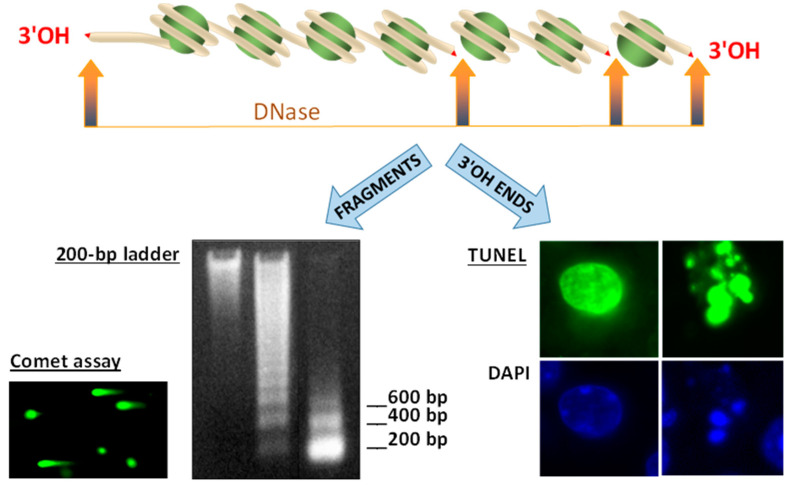
Three main DNA fragmentation assays: DNA ladder and comet assays vs. Table 200. bp apart from each other. The resulting double-stranded DNA fragments are seen as a nucleosomal 200-bp ladder in agarose gel. Comet assay is a single-cell variant of the same method, in which comet-like images represent DNA degraded in a dead cell, while dots without comets are formed by DNA from live cells. TUNEL labels 3′OH DNA termini in DNA within whole and fragmented nuclei.

Compared with two other primary methods of DNA fragmentation measurement, DNA ladder and comet assay, TUNEL has the advantage of being based on the identification of DNA termini rather than fragments (Figure 2). Theoretically, TUNEL should be more sensitive and linear for the identification of initial (low) DNA fragmentation than DNA ladder or comet, both of which have a lag-period of accumulation of small high-mobility fragments. In any case, studies combining TUNEL with either comet [41,42] or DNA ladder [43,44] have some advantage of catching early DNA fragmentation with maximal sensitivity.

## 3. TUNEL Applications

TUNEL is used to identify and quantify kidney injury in clinical and basic toxicological studies in a diverse range of applications, including medical treatments [45], environmental [46], agricultural [41] and industrial [47] exposures, and animal [48] and food sciences [49]. TUNEL is applicable to all cell types, organs, and species that have DNA and DNases, which includes just about all species. TUNEL has been used to identify and quantify kidney injury in a wide array of animals, including zebrafish [50], Japanese rice fish [51], chickens [48], gerbils [52], mice [41], rats [45], rabbits [53], mini-pigs [54], and humans [55]. An important advantage of TUNEL is that it can be used in fixed cultured cells as well as fixed tissues. This provides methodological consistency for comparison between in vitro and in vivo results in studies in which mechanisms can be investigated in cultured kidney cells, while in vivo implications are assessed in the animal kidneys. Anything in between whole cells and tissues can be used as well, including cultured cell spheroids [56] and ex vivo kidney slices [57,58].

**Figure 3:**
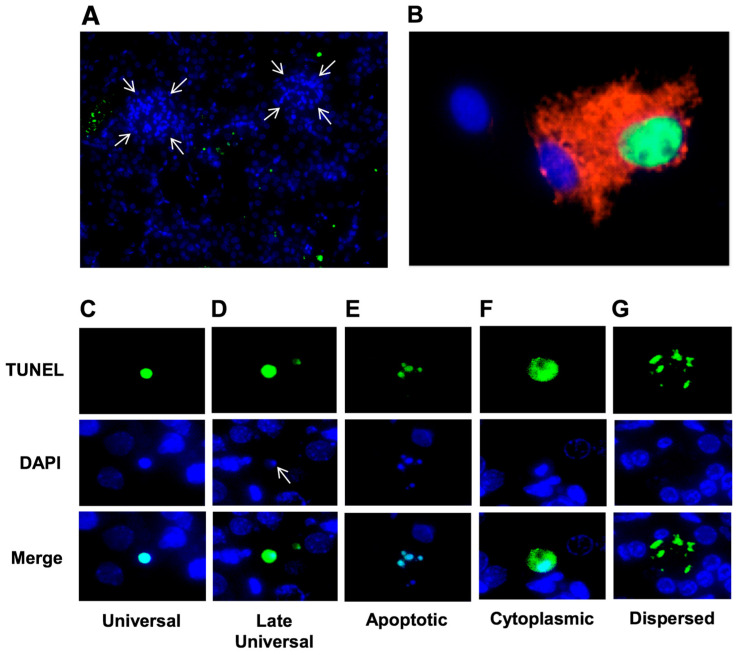
Variations of TUNEL images in kidneys of mice subjected to cisplatin injury (20 mg/kg, 4 days after exposure). (**A**) TUNEL image in kidney at low magnification; note poor TUNEL staining of glomeruli (shown by arrows) vs. tubules. (**B**) TUNEL-IHC combination; red staining indicates heme oxygenase 1 (HO-1) induced in two cells, one of which is TUNEL-positive (dead). (**C**) Commonly seen universal TUNEL signal indicative of any kind of cell death. (**D**) Late universal TUNEL signal with low DAPI staining of completely degraded DNA. (**E**) Apoptotic TUNEL signal; TUNEL staining of apoptotic bodies characteristic of apoptosis. (**F**) Cytoplasmic TUNEL signal that illustrates at least partial necrosis. (**G**) Necrotic dispersed TUNEL signal characterized by nuclear and plasma membrane lysis and irregular leakage of TUNEL-positive material from nucleus and cytoplasm. See Table 1 for a detailed description of TUNEL patterns shown in panels C through G.

The intensity of TUNEL in terms of the signal strength or the number of TUNEL-positive cells may vary between species depending on the activity of DNases. In our experience, rat kidney has more active DNase I than mouse kidney, and TUNEL-positive signal strength correlates with this. When the kidney is compared to other organs, its TUNEL-positive signal is very intense and similar to other organs with high DNase I activity, such as the intestine and salivary gland. On the other end of the spectrum, the lowest TUNEL-positive signal strength is observed in brain and tumor/cancer cells, where DNase I expression is most likely inactivated by alternative splicing in the coding region [59]. TUNEL-positive signal is observed in all cell types in the kidney. On average, tubular epithelium is damaged more often than glomeruli, especially in acute injuries (Figure 3A). This correlates well with the DNase I activity prevailing in these types of cells. Judging from sex and age differences observed in our experiments, DNase I is more abundant and active in males than females and mid-age animals compared to old or neonate-age animals. However, no clear-cut difference in TUNEL-positivity is evident in relation to sex or age, probably because the difference between these groups is not prominent or is predominated by other factors. In some kidney models (such as ischemia-reperfusion), DNase I is induced [9]; while in others, it is not and may even be suppressed (for example, in cisplatin toxicity) [10]. Activation of endonucleases, if present, has been shown to occur within the first 24 h after kidney injury [9,60,61]. Therefore, it was not surprising that at least half of the TUNEL applications in the kidney research in this review were used for acute kidney injury (AKI) since assessment of AKI is often made within 24 to 72 h after injury when endonucleases are most active. The list of AKI studies is long and includes toxic, septic, transplantation, and hypoxic injuries. In this work, we surveyed the models, exposure types, treatments, assessment timelines, and cell types affected in these studies. The studies that were surveyed covered acute and mixed acute-chronic modes of kidney injury and chronic kidney disease.

Whether kidney injury with TUNEL-positive signal is observed in tubules or the glomerulus depends on the type of damaging agent or disease. For example, lupus nephritis in humans and nickel nanoparticle damage was described as having strictly glomerular TUNEL-positive signals [62,63]. On the other hand, ischemia-reperfusion and streptozotocin-induced diabetic nephropathy in rats produced TUNEL-positive cells only in the tubules [64,65]. Proximal tubules, Henle’s loop, distal tubules, and collective ducts are often TUNEL-positive in ischemia-reperfusion models. TUNEL-positive cells in both the glomeruli and tubules were described in rats exposed to drugs such as cyclosporine A [66] and doxorubicin [67] or consumer product materials such as zinc oxide nanoparticles [68] and in a rat model of diabetes mellitus [69]. Septic shock-induced AKI in humans was also associated with both glomerular and tubular TUNEL-positive cells [70].

The most common causes of AKI are toxic exposure to drugs and sepsis. The most studied and commonly used drug toxicity model in kidneys is cisplatin AKI, perhaps because cisplatin produces a variety of molecular damages to kidney cells, including DNA and protein modification, membrane damage, and oxidative injury. In the kidney, the injury was localized to kidney tubular epithelial cells and peaked 72–96 h after cisplatin administration [71,72,73]. Other drugs known to induce AKIs include cyclosporine A [66], acetaminophen [74], tunicamycin [75], cyclophosphamide [76], gentamycin [77], and colistin [78]. Similar to cisplatin, TUNEL-positivity was observed in tubular epithelium in 3–7 days after administration of these drugs [66,78]. Other toxic AKIs induced by heavy metal (i.e., mercury) exposure [47,60], nanomaterial consumption [79,80], or associated with rhabdomyolysis [81] or contrast-induced nephropathy [54,82,83] resulted in TUNEL-positivity in tubular epithelium within hours and up to the first week after exposure. Sepsis-induced AKI is multifactorial and includes microcirculatory dysfunction and renal inflammation. Sepsis induced in rats and mice by lipopolysaccharide (LPS) endotoxin from bacterial capsules was strongly associated with TUNEL-positivity in proximal and distal tubules within 6–24 h after exposure [28,84,85,86]. Similar to the LPS-sepsis model, strangulated closed loop small bowel obstruction caused TUNEL-positivity in kidney in 3 h [87].

AKI can occur after transplantation and hypoxic injuries. Allograft rejection in humans is commonly associated with the elevation of TUNEL-positive cells [88,89]. After cold ischemia, TUNEL-positive cells were usually observed within 2–24 h during reperfusion [90], or more narrowly, between 14 and 17 h after ischemia [91], and sometimes were still observed as late as 7 days in transplant rejection [92]. Similarly, in ischemia-reperfusion models, TUNEL-positive cells were most often observed 24 h after reperfusion [44,93,94], or within the range between 4 [95,96] and 72 h [97]. In both transplantation and ischemic AKI studies, TUNEL-positive cells were localized to distal and proximal tubular epithelial cells [44,93]. In mouse kidney after ischemia-reperfusion injury, TUNEL-positive cells were described as concentrated in the corticomedullary junction, the usual target of ischemic injury [98].

Mixed acute-and-chronic injury, where toxic exposures, disease states, and/or assessments were both acute (i.e., hours, days) and chronic (i.e., weeks, months, years) in the same study, were observed in human transplant rejection (biopsy 1 week to 3 years post-transplant) [88], swine hepatitis E virus infection (7 and 14 days post-inoculation) [52], CO_2_ pneumoperitoneum-induced stress in hydronephrotic kidneys (2 weeks hydronephrosis, 2 days post-pneumoperitoneum) [53], mesangial proliferative glomerulonephritis induced by snake venom (1 to 14 days post-injection) [99], unilateral ureteral obstruction (1 to 14 days of obstruction) [100], uranyl acetate exposure (1 to 28 days of exposure) [101], aristocholic acid nephropathy (5 and 30-day daily exposure) [102], and neonatal hyperoxia (tested 1 to 60 postnatal days, exposed to hyperoxia first 14 days) [103]. Fourteen days after impact is a very common assessment time point for these kinds of kidney injuries [52,53,99,101,103]. In the majority of cases, TUNEL-positive cells were localized in tubules and collective ducts. The only exception was snake venom toxicity, which primarily damaged the glomeruli [99].

Chronic kidney disease (CKD), defined as the presence of kidney damage or decreased glomerular filtration rate (GFR) in humans for longer than 3 months, is also a common subject for TUNEL assessment, which can determine if CKD is associated with progressive kidney damage. The most commonly studied CKD in which TUNEL is applied is diabetic nephropathy. The injury identified by TUNEL-positivity occurred in tubules and podocytes [69] in diabetic patients with onset of diabetes prior to TUNEL testing ranging from 7 to 30 years [104,105] and 2 to 16 weeks after streptozotocin treatment in rodents [106,107]. Similar TUNEL-positive histopathology was reported in immunoglobulin A nephropathy [22]. In lupus nephritis and membranoproliferative glomerulonephritis, TUNEL-positivity was seen primarily in the glomeruli [62,108,109]. On the other hand, drug safety studies of cisplatin [110], chloroquine [45], doxorubicin [67], finasteride [23], tacrolimus [111], and levetiracetam [24] observed mostly tubular damage. Likewise, the continued acute injury identified by tubular TUNEL-positive cells were described in studies of uric acid nephropathy [112], methionine deficiency [48], exposure to environmental toxins microcystin-LR [50] and cadmium [113], and in association with calcium-oxalate induced kidney stones [114] and calcifying nanoparticles [115]. While identification of the progressive kidney damage during CKD by using TUNEL assay is a useful diagnostic tool, combining TUNEL assay with immunohistochemistry (IHC) would perhaps be even more informative in determining the mechanism of the injury.

## 4. TUNEL Quantification and Colocalization Techniques

Methods of TUNEL quantification vary. In the majority of reports, TUNEL-positive cells/nuclei are quantified as the percent of total cells/nuclei [83,116,117]. This seems the right thing to do, although the number of TUNEL-positive cells likely do not correspond exactly to the number of dead cells. Some cells may not have reached identifiable levels of DNA fragmentation, while others could already have been eliminated by macrophages. Quantification of the number of TUNEL-positive cells per square unit (mm^2^) is also used [118], but this method seems less precise because it depends on the location and cell type content of the areas. Cortical sections contain mostly glomeruli, tubules, blood vessels, and medullary rays, while medullary sections primarily contain loops of Henle, collecting ducts, and blood vessels. Quantification “per field” certainly depends on what the field is [72,119], and quantification “per section” is even more undefined [61,120]. Poorly identified measurements, such as “histological score” [121], “apoptotic index” [113], “apoptosis rate” [92], “percentage of apoptosis” [122], or percentage of untreated control [123,124], seem to be even less appropriate, and make interpretation difficult and not comparable with the majority of studies.

Cell death in neighboring cells starts unevenly because it depends on vascularization, cell cycle, and other variables. This often results in uneven TUNEL staining of cells in the tissue [45,96,125]. Surprisingly, the general intensity of the TUNEL-positive signal is not used for TUNEL measurement, despite it directly depending on the degree of DNA fragmentation. This is perhaps because cells are considered dead independent of the intensity of the TUNEL signal, as a cell cannot be “more dead” if the TUNEL staining is more intense. Although it is difficult to compare studies that use a wide variety of methods to quantify TUNEL results, the amount of TUNEL-positive cells in control animals is usually below 2% [75,99,100,116,126]. Acute injury is associated with a ~5–40× increase in TUNEL-positivity [79,126,127]. During chronic injury, the increase in TUNEL-positive cells is slightly lower, ~2–20-fold, [29,43,65] compared to untreated controls.

The initial TUNEL assay based on diaminobenzidine (DAB) staining was designed for light microscopy [14,15]. This traditional method was still used in two-thirds of the studies surveyed in this review and in the overwhelming majority of human biopsy studies. The quantification is done by manual cell count and, while precise, it is time-consuming. Often, quantification is not used at all, and the results are presented using images of treated and stained samples as simple evidence that cell death took place. Automation may speed up the quantification process [128,129]. However, DAB cannot be combined with any other staining in the same sample, and thus DAB-based TUNEL can only provide the number of dead cells, without any identification of the cell type (except visual recognition) or cell death mechanism.

While human studies overwhelmingly rely on DAB-based TUNEL using light microscopy, approximately half of animal studies use fluorescent microscopy (Figure 3). Fluorescent TUNEL is visually attractive, easily interpretable, and has several major advantages. In addition to precise quantification by identification of TUNEL-positive nuclei or mean intensity of the color, fluorescent TUNEL signal can be colocalized with other colors for histochemistry (e.g., DAPI) (Figure 3A) or IHC (Figure 3B). In the latter, the use of protein markers may help to identify TUNEL-positive signals associated with different kidney compartments or give information on the type of cell death. We could not find any examples of TUNEL combined with double IHC antibody staining. While such a combination would be very informative, for example, for the identification of a cell death mechanism in certain cell types, the technical difficulties associated with such staining may outweigh the benefits.

Identification of kidney cell type is certainly not a problem for an experienced kidney pathologist [104]. Because of this, the use of additional markers for cell-type identification, for example, using CD31 for the identification of endothelial cells [130], is rare. However, the use of IHC protein markers for the characterization of tissue injury is often used. Most studies use TUNEL-positivity as the sole evidence of apoptosis. In other studies, recognition that TUNEL-positivity is not specific for apoptosis has led to many morphological (e.g., hematoxylin and eosin or periodic acid–Schiff staining), biochemical, and IHC markers to be combined with TUNEL staining to confirm apoptosis. The most commonly used marker for caspase-dependent apoptosis is cleaved (active) caspase-3 by using an antibody that does not recognize the full-size caspase-3 [131,132]. Both TUNEL and cleaved caspase-3 assays showed exclusively tubular, not glomerular, staining in rat ischemia-reperfusion [64]. Other caspase studies used caspase-1 [27], -9 [53], -11 [28], and -12 [75]. Other apoptotic markers included the B-cell lymphoma 2 (Bcl2) family of proteins Bcl-2 [27], Bcl2L1 [43], Bcl-xl [71], Bax [99], Bak [49], and Bad [43]; apoptotic receptor Fas [52] and apoptotic receptor ligand FasL [66]; forkhead box (FOX) protein transcription factors Fox01 [133] and Fox03 [27]; and cytochrome c [53]. Inflammation was identified by IHC of tumor necrosis factor alpha (TNFα) [134]; tumor growth factors TGFβ [66] and TGF-β1 [135]; serine/threonine kinase 1 (SGK1) [135]; electron-transfer flavoprotein, beta-subunit (ETFβ) [136]; Janus kinase-2 (JAK2) [137]; signal transducer and activator of transcription 3 (STAT3) [137]; nuclear factor kappa-light-chain enhancer of activated B cells (NF-κB) [137] and its inhibitor protein IκB [137]. For kidney injury assessment, kidney injury molecule-1 (KIM-1) [24], aquaporin-1 channel (AQP-1) [45], hypoxia-inducible factor 1-alpha transcription factor (HIF-1α) [103], Asc-type amino acid transporter-1 (Asc-1) [121], mammalian target of rapamycin (mTOR) [131] kinase, and transglutaminase II [89] were used. Oxidative kidney injury was studied using markers such as 8-hydroxyguanosine (8OHdG) [102], neutrophil gelatinase-associated lipocalin (NGAL) [102], NAD-dependent deacetylase sirtuin-1 (SIRT1) [138], and heme oxygenase 1 (HO-1) [90]. Kinases were intensively studied, including c-Jun N-terminal kinase (JNK) pathway [101], phosphoinositide 3-kinase/protein kinase B/nuclear factor erythroid 2-related factor 2 transcription factor (PI3K/Akt/Nrf2) pathway [46], pannexin-1 (PANX1) plasma membrane gap junction protein [26], and mitogen-activated protein kinase/extracellular signal-regulated kinase (MAPK/ERK) [26]. Studies of apoptotic endonucleases involved caspase-activated DNase (CAD) [139], DNase I [108], and EndoG [139], and DNA damage markers included tumor protein p53 (p53) [99] and dynamin-related protein 1 (PARP1) [66]. Some studies also found the assessment of endoplasmic reticulum stress-mediated apoptosis and tissue injury useful [75].

Given the large number of mechanistic studies surveyed and the advantage of combining TUNEL with IHC markers for the identification of cell death mechanisms, one would expect many studies using colocalization between the two. The analysis may be performed by a pixel-by-pixel colocalization between TUNEL-positive cells and IHC markers, IHC marker mean intensity in TUNEL-positive areas, or TUNEL-positive signal mean intensity in IHC marker-positive areas. Unfortunately, this advantage of TUNEL has been used in only a few studies. TUNEL-positive cells were colocalized with cleaved caspase-3 in kidney tubules during acetaminophen toxicity in rats [74]. IHC colocalization with TUNEL-positive signal allowed cell death identification of mesangial cells during Habu nephritis [99]. In light microscopy, colocalization with mechanistic markers of toxicity was done in serial sections, as reported by Ott et al. [89].

## 5. TUNEL Problems and Limitations

Several potential problems can take place and should be expected when working with TUNEL. One is that 3′OH DNA termini occur not only during cell death as a result of endonuclease action but also as a normal intermediate metabolite in almost all enzymatic reactions with DNA. A primary cell function that produces 3′OH termini is DNA synthesis, in which numerous Okazaki fragments may theoretically produce a false-positive TUNEL signal, especially in highly-sensitive modifications of the assay. During kidney injury, DNA repair may also contribute to false TUNEL-positive signals as a result of an AP-endonuclease action. Massive oxidative kidney injury induced by chemical (hydrogen peroxide, bleomycin) or physical (gamma irradiation) injury may add to the number of 3′OH ends, increasing the false-positive TUNEL readings apart from endonuclease-mediated DNA fragmentation.

More often and more likely, improperly set experimental conditions of the TdT reaction (time, temperature, enzymatic activity, or cofactors) may result in an artificially elevated TUNEL-positive signal background. Although it remains undefined as to what is considered the normal baseline for untreated kidney tissue samples, it seems plausible that around 1% of total cells would be a reasonable number. Certainly, a 10% or higher background is clear evidence of either uncharacterized and unrefined assay conditions or a deviation from appropriate sample storage conditions.

Another problem to expect is low DNase I activity in some cell types (e.g., endothelium), or kidney compartments (e.g., glomeruli), or pathological tissues (e.g., tumors). These cells should be expected to show low TUNEL positivity, not because they are injury-resistant, but because there is not enough endonuclease activity to produce a measurable signal in TUNEL. In our experience, even changes between 0.01% and 0.1% of TUNEL-positive cells can be statistically significant and used as solid evidence of kidney (of another tissue) injury.

Finally, a problem with quantification of TUNEL staining arises when the amount of DNA fragmentation is too high, for example, at late stages of cell death or in high-intensity injuries, where remaining DNA cannot be properly counterstained with DAPI due to disassembly of the double helix (Figure 3D). In these cases, TUNEL-positive signals are “hanging in the air”, and seemingly not associated with a nucleus. It becomes the responsibility of a pathologist to interpret these as TUNEL-positive cells rather than an artifact. With fluorescein-labeled TUNEL, there is a danger of identifying background autofluorescence in tissue as TUNEL-positivity [140]. In these cases, a switch to red spectrum TUNEL [51,54] is recommended.

## 6. TUNEL Patterns as a Source of Additional Information

Several TUNEL patterns can be clearly distinguished and used to determine a potential mechanism and degree of injury (Table 1, Figure 3). Usually, a nuclei-only type of TUNEL pattern is produced by mild injury, while stronger injury (i.e., elevated exposure) results in stronger staining and disintegration of nuclei or cells.

A strictly apoptotic TUNEL pattern is nuclear fragmentation as a result of apoptotic bodies formation (Figure 3E). Small fragments of TUNEL-positive nuclei, which are likely apoptotic bodies, were seen in rat ischemia-reperfusion at 24–72 h [97]. Apoptotic bodies were also seen with TUNEL labeling of nuclei in the renal tubular epithelium and interstitium 14 days after ischemia-reperfusion injury in rats [141]. Increasing doses of lavender oil reduced nuclei-only TUNEL patterns and converted them to necrosis-like TUNEL with cytoplasmic leakage [94]. The latter was a result of nuclear envelope destruction due to the action of proteinases and lipases in necrosis. Cytoplasmic TUNEL (Figure 3F) seen as a TUNEL-positive cytoplasm was observed in light microscopy in rhabdomyolysis model in rat tubules [142] and in podocytes cultured in the presence of high glucose and in diabetic nephropathy in mice [135]. Cytoplasmic TUNEL was also described in renal ischemia-reperfusion in mice using fluorescent TUNEL [98] and in ischemia-reperfusion in rats using light microscopy [143]. Mercuric chloride induced profound and exclusively tubular damage with strong cytoplasmic TUNEL staining indicative of nuclear membrane damage [60]. If the injury continues or the dose of the damaging agent increases, cytoplasmic TUNEL can be converted to a “messy” picture of dispersed TUNEL (Figure 3G) attributed to late stages of cell destruction due to necrosis and spillage of cellular cytoplasm and debris. For example, both cytoplasmic and dispersed TUNEL was observed in cisplatin-treated mice [71,72] and rats [144], and in podocyte injury in streptozotocin-induced diabetic rats [145]. Dispersed necrotic TUNEL was also observed during uranyl acetate-induced nephrotoxicity in mice [101]. Lastly, another TUNEL signal variable to pay attention to is the intensity of TUNEL-positive signals, which may deviate from cell to cell depending on the number of breaks (degree of injury) each cell experiences, as documented by some studies [45,96,125]. For models in which varying TUNEL signal intensity is observed, quantification by both the number of TUNEL-positive objects and the mean intensity of the staining is recommended.

## 7. Conclusions

Due to the high activity of apoptotic endonucleases in the kidney, particularly DNase I and EndoG, the use of TUNEL for kidney research and diagnosis is very informative. It may become even more useful if TUNEL is applied beyond its initial use as a method to identify apoptosis. Applying TUNEL exclusively for apoptosis studies is a good example of how science can be inadvertently misdirected by an inappropriately used method. Despite its use as a mostly acute injury assay, TUNEL can be successfully applied to assess both AKI and CKD. However, in CKD, the degree of TUNEL-positivity should be expected to be lower than in acute injury. Because the activity of endonucleases (mainly, DNase I) is much higher in tubular epithelium than in other kidney compartments, injury to kidney tubules is usually seen as more prominent. However, glomerular or vascular injuries can also be studied using TUNEL if the injury actually occurs in these compartments. The colocalization of fluorescent TUNEL with IHC markers permits the identification and association of cell types and cell death mechanism with TUNEL signals. TUNEL quantification can contribute valuable information on the degree of cell death, and applying image analysis software can be very useful in this regard. Considering the variety of TUNEL patterns, representative images are strongly suggested to establish and demonstrate TUNEL signals that are considered positive. Finally, TUNEL patterns should be used as a source of additional information, such as the mechanism of cell death and the degree of toxicity. For example, distinguishing between TUNEL-positive apoptotic bodies, the presence of cytoplasmic TUNEL signals, or dispersed TUNEL patterns may help identify apoptotic vs. necrotic TUNEL-positive cells for more precise interpretations of research and diagnostics results.

## Figures and Tables

**Table 1:** TUNEL patterns (images are shown in Figure 3C–G).

	TUNEL Image	DAPI Image	Mechanism	Type of Cell Death
**Universal TUNEL**	Round or oval TUNEL-positive object the size of an average nucleus	Round or oval object of equal size	Large DNA fragments inside nuclear envelope	**Any**
**Late Universal TUNEL**	Round or oval TUNEL-positive object the size of an average nucleus	Round or oval object of equal size, or no DAPI-positive object at all	Small DNA fragments inside nuclear envelope	**Any**
**Apoptotic TUNEL**	A group of round objects of varying sizes located near each other	A group of objects of the same size and shape as TUNEL-positive objects	Apoptotic bodies: fragmented nucleus containing large DNA fragments	**Apoptosis**
**Cytoplasmic TUNEL**	TUNEL-positive object the size of the entire cell; cytoplasmic TUNEL may have less intensity than nuclear TUNEL	Nucleus is smaller than the TUNEL-positive area	Leakage of small DNA fragments to cytoplasm through damaged nuclear envelope	**Necrosis**
**Dispersed TUNEL**	Irregular TUNEL-positive objects and “spills” of TUNEL-positive material	DAPI staining is negligent in the areas of the TUNEL objects	Leakage of DNA small fragments through damaged nuclear envelope and plasma membrane	**Necrosis**

## Supplementary Materials

Supplementary Materials can be found at https://www.mdpi.com/1422-0067/22/1/412/s1.
