# Supplementary material for: TUNEL Assay: A Powerful Tool for Kidney Injury Evaluation
Source: Int J Mol Sci. 2021 Jan 2;22(1):412. doi: 10.3390/ijms22010412 (PMC7795088; doi:10.3390/ijms22010412)
Supplement: Supplementary file 1 [file ijms-22-00412-s001.pdf]

SUPPLEMENT

FIGURE

TUNEL Assay: A Powerful Tool for Kidney Injury Evaluation

Christopher L. Moore,[1] Alena V. Savenka,[1] and Alexei G. Basnakian[1,2]

[1] Department of Pharmacology & Toxicology, University of Arkansas for Medical Sciences, 4301 West Markham Street, #638, Little Rock, AR 72205, USA

[2] John L. McClellan Memorial VA Hospital, Central Arkansas Veterans Healthcare System, 4300 West 7th Street, Little Rock, AR 72205, USA

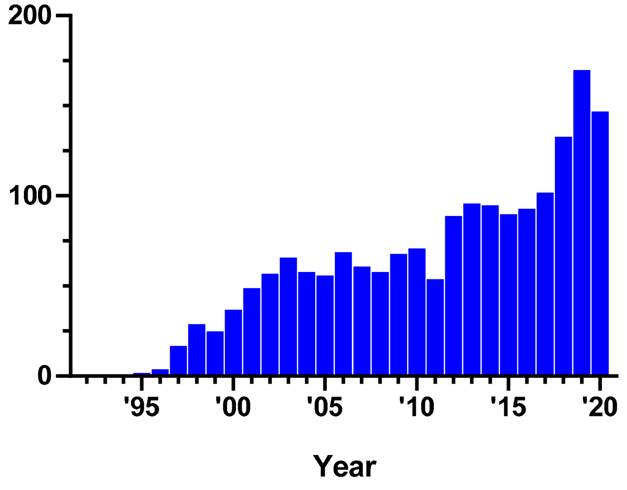

Supplement Figure S1. The number of kidney-related peer-reviewed articles using TUNEL assay published per year in the period between 1992 and December 10, 2020.
